# Trait Empathy Shapes Neural Responses Toward Sad Music

**DOI:** 10.3758/s13415-020-00861-x

**Published:** 2021-01-20

**Authors:** Liila Taruffi, Stavros Skouras, Corinna Pehrs, Stefan Koelsch

**Affiliations:** 1 Department of Music, Durham University, Durham, UK; 2 Department of Biological and Medical Psychology, University of Bergen, Bergen, Norway; 3 Bernstein Center for Computational Neuroscience, Charité – Universitätsmedizin, Berlin, Germany

**Keywords:** Trait empathy, Sad music, Social neuroscience, Music & emotion, fMRI

## Abstract

Individuals with a predisposition to empathize engage with sad music in a compelling way, experiencing overall more pleasurable emotions. However, the neural mechanisms underlying these music-related experiences in empathic individuals are unknown. The present study tested whether dispositional empathy modulates neural responses to sad compared with happy music. Twenty-four participants underwent fMRI while listening to 4-min blocks of music evoking sadness or happiness. Using voxel-wise regression, we found a positive correlation between trait empathy (with scores assessed by the Interpersonal Reactivity Index) and eigenvector centrality values in the ventromedial prefrontal cortex (vmPFC), including the medial orbitofrontal cortex (mOFC). We then performed a functional connectivity (FC) analysis to detect network nodes showing stronger FC with the vmPFC/mOFC during the presentation of sad versus happy music. By doing so, we identified a “music-empathy” network (vmPFC/mOFC, dorsomedial prefrontal cortex, primary visual cortex, bilateral claustrum and putamen, and cerebellum) that is spontaneously recruited while listening to sad music and includes brain regions that support the coding of compassion, mentalizing, and visual mental imagery. Importantly, our findings extend the current understanding of empathic behaviors to the musical domain and pinpoint sad music as an effective stimulus to be employed in social neuroscience research.

## Introduction

Empathy is one of the most remarkable human abilities that allows, for instance, to understand what it feels like to experience someone else’s joy or sadness and that ultimately promotes meaningful social interaction. Empathic behaviors are diverse and include resonating affectively with others’ emotions (*affective empathy* or *experience sharing*) and comprehending others’ mental and affective states (*cognitive empathy* or *mentalizing*) (Zaki & Ochsner, 2012). While sharing another person’s positive emotion is doubtlessly pleasant, shared negative affective experiences can be challenging and may lead to *empathic distress*, a maladaptive empathic response that is associated with burnout in individuals who are routinely exposed to the suffering of others, such as physicians, nurses, and therapists (McCray et al., 2008). *Compassion* represents an alternative empathic reaction to sharing a negative emotion. In contrast to empathic distress, compassion is characterized by positive feelings of warmth and care as well as strong prosocial motivation and approach components (Singer & Klimecki, 2014). Research on the neural underpinnings of affective empathy has predominantly focused on empathy for physical pain, revealing that the medial/anterior cingulate cortex and the anterior insula are consistently activated during first-hand experience of pain as well as while observing another person in pain (Jackson et al., 2005; Lamm et al., 2010; Singer et al., 2004; for a meta-analysis see Lamm et al., 2011). Although the neuroimaging literature on compassion is limited compared with the one on empathy for pain, a number of studies have underlined the role of nonoverlapping brain structures, such as the medial orbitofrontal cortex (mOFC), putamen, pallidum, and ventral tegmental area, extending the dissociation between empathic distress and compassion from the psychological to the neural level (Klimecki et al., 2012; Klimecki et al., 2013). With regard to cognitive empathy, the dorsomedial prefrontal cortex (dmPFC), the superior temporal sulcus/temporoparietal junction, the posterior cingulate cortex, and the temporal poles are reliably engaged when participants are asked to make judgments about targets’ beliefs, thoughts, intentions, and emotions (Denny et al., 2012; Mitchell, 2009; Pehrs et al., 2018; Preckel et al., 2018; Van Overwalle & Baetens, 2009).

Although social neuroscientists have traditionally investigated empathy as an interpersonal phenomenon directed to human beings (e.g., Lamm et al., 2010), empathic responses also extend to aesthetic contexts (i.e., imagined affective experiences, beliefs, or intentions of inanimate characters of the artwork or of the artist) and, in fact, empathy is considered to be a constitutive element of the aesthetic experience in cinema (D’Aloia, 2012), figurative art (Freedberg & Gallese, 2007), literature (Johnson et al., 2013), and music (Levinson, 1997). As argued by the philosophers Robert Vischer (1873) and Theodor Lipps (1903), appreciation of art draws crucially on the beholders’ ability to resonate with the piece of art, thereby underlining the importance of individual characteristics and suggesting that highly empathic individuals may have more intense and pleasurable aesthetic experiences. These philosophical accounts of “aesthetic” empathy have stressed its affective component rather than the cognitive one. However, mentalizing processes also are at play in aesthetic contexts, such as music listening. Similar to other art forms, music is the product of—and therefore is expressive of—human feelings, but also beliefs, and intentions. By conveying meaning and signaling inferred intentions, music can act as social agent (or virtual surrogate for social interaction; Schäfer & Eerola, 2018), even in the absence of any perceptual information indicating the presence of a human agent (Livingstone & Thompson, 2009). In line with this perspective, a previous fMRI study, comparing man-made music versus music that participants believed to have been generated by a computer, demonstrated that the former recruits brain areas involved in the attribution of mental states or mentalizing, such as the dmPFC (Steinbeis & Koelsch, 2009). These empirical findings add to the theoretical accounts of music as a stimulus with social significance and call for a more systematic investigation of the neural correlates of empathy-related processes (both affective and cognitive) in musical contexts (Clarke et al., 2015). In particular, sad-sounding music (henceforth referred to as “sad music”) may act as a powerful and sophisticated “social” stimulus to map the empathic brain, being capable to evoke multifaceted, yet mostly pleasurable, intense affective experiences despite sadness being a negative emotion (Eerola et al., 2018; Sachs et al., 2015).

On the behavioral level, music-and-emotion studies have provided mounting evidence, yet merely correlational in nature and limited by the use of convenience samples, of a close relationship between empathy disposition and enjoyment as well as sensitivity to sad music. Specifically, individuals who score high on self-report questionnaires of empathy, the most widely used scale being the Interpersonal Reactivity Index (IRI; Davis, 1980), experience more intense emotions, a feeling of “being moved”, and enjoyment while listening to sad music compared with individuals who score low (Eerola et al., 2016; Garrido & Schubert, 2011; Kawakami & Katahira, 2015; Taruffi & Koelsch, 2014; Vuoskoski & Eerola, 2012; Vuoskoski et al., 2012). The association between trait empathy and sad music draws not only on emotional but also cognitive aspects of empathy, as indicated by the positive correlation between the empathy subscale *fantasy* of the IRI (which assesses the tendency to transpose one’s self imaginatively into the feelings and actions of fictitious characters in books, movies, and plays; Davis, 1980) and the liking as well as the intensity of sad music (Taruffi & Koelsch, 2014; Vuoskoski et al., 2012). These findings suggest that sad music may trigger empathic listeners to fantasize about mental images related to the unfolding of the music. Interestingly, this is in line with recent evidence that sad (compared with happy) music is associated with higher levels of mind-wandering in the form of visual mental images and the engagement of the default mode network (Taruffi, Pehrs, et al., 2017), which overlaps to a great extent with the abovementioned core regions involved in mentalizing (e.g., Mars et al., 2012). Only one previous fMRI study investigated the neural substrates underlying the relationship between trait empathy and music, providing evidence that individual variance in trait empathy is reflected in differential recruitment of core empathy networks during music listening (Wallmark et al., 2018). Specifically, IRI subscales were found to correlate with activity in regions associated with both emotional (sensorimotor regions, insular, and cingulate cortex) and cognitive empathy (prefrontal cortex and temporoparietal junction) during passive listening tasks. However, this study featured only simple musical tones and 16-s music excerpts varying in familiarity (familiar and unfamiliar) and preference (liking or disliking), but not in emotional tone (e.g., sad or happy). Therefore, it remains to be tested yet whether empathic participants are particularly sensitive to sad music, showing specific empathy-related brain activity patterns.

The present study sought to investigate whether empathic abilities relate to variation in brain network connectivity in response to sad (vs. happy) music. Participants were scanned while listening to 4-min blocks of sad and happy music and subsequently completed the IRI (Davis, 1980). Functional data were analyzed using Eigenvector Centrality Mapping (ECM; Lohmann et al., 2010) and Functional Connectivity (FC), similar to a previous study that investigated “small-world” networks underlying music-evoked joy (Koelsch & Skouras, 2014). ECM is a mathematical method that has been described in detail previously (Lohmann et al., 2010). For interpretational purposes, ECM derives a measure of Eigenvector Centrality (EC) for each voxel within a brain volume, based on timeseries data from each and every other voxel within the same brain volume. Through an iterative self-referential procedure, ECM considers the patterns of interconnectivity across all voxels. The derived EC values correspond to the level of influence that each voxel exerts over the entire brain volume activation pattern. Note that EC values do not only take the number of connections of a voxel into account, but also the importance of connected voxels. For instance, Google's PageRank algorithm was based on EC, such that web domains were not only ranked higher when more other pages linked to them, but also when those linking pages themselves were pages to which more other pages linked. Thus, ECM can reveal influential, or important, hubs of neural networks in the human brain. In this study, first a second-level regression analysis was performed voxel-wise, with IRI scores as the predictor and EC values from the contrasts between emotion conditions as the outcome variable. Then, the cluster identified by this analysis was used as seed region in a subsequent FC analysis that was performed to identify a network of brain regions underlying empathic individuals’ responses evoked by sad compared with happy music. We expected to observe the engagement of brain regions that are involved in compassion and positive affect, in particular the mOFC (Klimecki et al., 2012; Klimecki et al., 2013). This hypothesis was motivated by the fact that empathic individuals show an enhanced enjoyment of sad music, suggesting that they exhibit patterns of empathic reactions to negatively valenced stimuli that are more aligned with compassion rather than emotional distress. In addition, given the association between cognitive aspects of dispositional empathy and the liking of sad music (Taruffi & Koelsch, 2014; Vuoskoski et al., 2012), we anticipated the engagement of mentalizing brain regions, specifically the dmPFC, because of its role in social inferences of traits and scripts about other people (Van Overwalle, 2009). Moreover, the recruitment of the dmPFC has previously been observed in music studies exploring: (*i*) neural associations with trait empathy (Wallmark et al., 2018); (*ii*) internally oriented cognitive experiences, such as mind-wandering in response to sad music (Taruffi, Pehrs, et al., 2017); and (*iii*) attribution of mental states (Steinbeis & Koelsch, 2009). We finally expected that trait empathy would be associated with activity in the visual cortex, given that previous findings suggested enhanced visual mental imagery processes during listening to sad music (Taruffi, Pehrs, et al., 2017).

## Methods

### Participants

Twenty-four (12 females) right-handed, native German speakers with no history of neurological problems participated in this study (mean age = 25.3, age range 21-34). Participants were screened for depressive symptoms, alexithymia (alexithymia is associated with difficulties in perception of sadness conveyed by music; Taruffi, Allen, et al., 2017), and sensitivity to music reward, using the Quick Inventory of Depressive Symptomatology (QIDS-SR; Rush et al., 2003), the Toronto Alexithymia Scale (TAS-20; Bagby et al., 1994), and the Barcelona Music Reward Questionnaire (BMRQ; Mas-Herrero et al., 2013), respectively. All participants scored below 6 on the QIDS-SR and 52 on the TAS-20; thus, none of the participants were depressive or alexithymic. With regard to the BMRQ, all participants scored between 40 and 60 on the two factors of *emotion evocation* and *mood regulation*, indicating an average sensitivity to reward derived from music-evoked emotional experiences. None of the participants were professional musicians. 58.3% of the participants were nonmusicians, 29.2% amateur musicians, and 12.5% semiprofessional musicians. Participants’ favorite musical genres fell into the following categories: 25.7% rock, 20% electronic, 15.7% pop, 15.7% classical and soundtrack, 12.8% jazz, 5.7% reggae, and 4.4 % other. All participants provided informed consent in a manner approved by the Ethics Committee of the Freie Universität Berlin, and the experiment was performed in accordance with ethical standards outlined by the Declaration of Helsinki. Participants either received course credit or 10€/h for participation.

### Music stimuli

The stimulus set consisted of four pairs of sad-happy excerpts of instrumental film soundtracks, capable of evoking sad and happy emotions, respectively. Each sad-happy pair had the same tempo (measured in beats per minute, BPM) and featured an acoustically identical beat track, leading to the same perceived tempo and similar vestibular responses for sad and happy music (for more information about the stimulus preparation see Taruffi, Pehrs, et al., 2017). There were four “short” (35–37 s) and four “long” (1.18–1.30 min) excerpts, counterbalanced across conditions. All excerpts were edited to have 1.5-s fade in/out ramps and were RMS (root mean square) normalized to have the same loudness. Stimuli of the same emotion category were concatenated into blocks of 4-min duration (no stimulus was repeated) to ensure optimal data for the application of ECM analysis, which typically requires relatively long trial periods but has the advantage that only one trial per condition is sufficient per subject (Lohmann et al., 2010).

### Self-report measure of trait empathy

Individual differences in trait empathy were measured through the validated German version (Paulus, 2009) of the IRI (Davis, 1980). The IRI is one of the most commonly used self-report questionnaires of dispositional empathy, which builds on a multidimensional conceptualization of empathy, including cognitive and affective aspects, and has been consistently used in previous studies that examined the relationship between sad music and trait empathy (e.g., Vuoskoski et al., 2012). Global empathy scores showed a *M* of 15.4 and a *SD* of 1.37 (corresponding *M* of German population norms = 14.49, and *SD* = 3.17; Paulus, 2009).

### Procedure

Because familiarity can strongly affect music-evoked emotions and their neural correlates (Pereira et al., 2011), approximately two weeks before the scanning session the participants were tested on their familiarity with the music stimuli to ensure that they were unfamiliar with the selected music excerpts. Participants listened to short excerpts (15 s) of the stimuli and indicated their familiarity with each excerpt on a scale ranging from 1 (“I have never heard this piece before”) to 5 (“I know this piece”). Participants were not included in the fMRI session if they were familiar with any of the music pieces. A paired *t*-test showed that there was no significant difference in familiarity between the happy [1.62 ± 0.57 (*M* ± *SD*)] and the sad pieces (1.57 ± 0.63), *P* > 0.05.

In the scanning session, participants listened to the 4-min sad and happy music blocks presented in a pseudo-randomized order. Stimuli were presented via MRI-compatible headphones (under which participants wore earplugs) at a comfortable volume level and participants were instructed to close their eyes and relax during the music listening. Each music block was followed by: (*i*) a 2-s signal tone indicating participants to open their eyes; (*ii*) a 16-s evaluation period during which participants were asked to indicate their overall emotional state during the 4-min music period using a response pad they held in their right hands; and (*iii*) a 10-s silence period to avoid emotional crossover between different blocks of stimuli.

For the emotion evaluation in response to the music, participants were instructed to focus on their emotional experience (i.e., *felt emotions*) rather than the emotional tone that the music was intended to convey (i.e., *perceived emotion*). We decided to assess only *felt emotions*, because the link between trait empathy and sad music has been reported mainly on experiential rather than perceptual level (e.g., Vuoskoski, & Eerola, 2012). Furthermore, no clear findings are available to substantiate the claim that behavioral differences between felt and perceived emotions correspond to separate underlying neural correlates (e.g., Koelsch, 2014). Participants rated their felt emotions on four 6-point scales representing valence (“How unpleasant/pleasant did you feel during the music listening?”), arousal (“How calm/aroused did you feel during the music listening?”), sadness (“How sad did you feel during the music listening?”), and happiness (“How happy did you feel during the music listening?”). The answer scales ranged from 1 (“very unpleasant”, “very calm”, “not at all”) to 6 (“very pleasant”, “very aroused”, “very much so”).

The total length of the fMRI session was approximately 27 min and, besides the two experimental conditions, included also listening to two blocks of dissonant and neutral music as well as a resting state scanning session with no music (these scans were acquired to fulfill other research purposes). All 24 participants completed the IRI after the scanning session.

### fMRI data acquisition and data analysis

MRI data were acquired on a 3T Siemens Magnetom Trio MRI scanner, at the Dahlem Institute for Neuroimaging of Emotion. Before functional scanning, a high-resolution (1 × 1 × 1 mm) T1-weighted anatomical reference image was obtained from each participant using a rapid acquisition gradient echo (MP-RAGE) sequence. Functional data were acquired using a continuous echo planar imaging (EPI) sequence (37 slices interleaved; slice thickness = 3 mm; interslice gap = 0.6 mm; TE = 30 ms; TR = 2,250 ms; flip angle = 70°; matrix = 64x64; FOV = 192 x 192 mm). To minimize susceptibility artifacts in areas, such as the orbitofrontal cortex and the temporal lobes, the acquisition window was tilted at an angle of 30° to the intercommissural (AC-PC) plane (Deichmann et al., 2003; Weiskopf et al., 2007), similar to previous studies (Koelsch et al., 2013; Koelsch & Skouras, 2014).

Functional images were preprocessed and analyzed using LIPSIA 2.1 (Lohmann et al., 2001). Each participant’s anatomical T1 data were used to derive nonlinear transformation matrices between the participant’s native space and MNI-space. Data were corrected for slicetime acquisition, realigned and normalized, by applying the derived transformation matrices, into MNI-space-registered images with isotropic voxels of 3 mm^3^. A high-pass filter with a cutoff frequency of 1/90 Hz was used to remove low-frequency drifts in the fMRI time-series, and a spatial smoothing was performed using a Gaussian kernel of 6 mm full-width at half-maximum. The mean signal value per scanned volume was computed and regressed out of each participant's data. To control for motion artifacts, the movement parameters of each participant also were regressed out of the respective fMRI time-series.

Whole-brain EC maps were computed separately for each participant during each 4-min experimental condition. Global empathy scores were used as the regressor of interest, with age and gender as covariates of no interest (as in Koelsch et al., 2013), in a second-level design matrix comparing EC between the two experimental conditions using voxel-wise paired sample *t*-tests. Regression was used, because empathy scores were normally distributed, as confirmed by a Kolmogorov-Smirnov test, D(24) = 0.16, *P* > 0.05. Results of this multiple regression analysis were corrected for multiple comparisons using cluster-size and cluster-value thresholds obtained by Monte Carlo simulations with a significance level of *P* < 0.05 (Lohmann et al., 2008).

FC analysis was conducted using as seed region the cluster identified by the above-described voxel-wise regression analysis between EC and empathy scores. The average time-course of activity within the seed region was extracted and regressed against activity in the rest of the brain, separately for the sad and happy music conditions. FC maps were first computed separately for each participant and later normalized across the whole sample. Then, FC maps were compared between the two experimental conditions using paired sample *t*-tests corrected for multiple comparisons (using cluster-size and cluster-value thresholds obtained by Monte Carlo simulations with a significance level of *P* < 0.05; Lohmann et al., 2008).

## Results

### Behavioral results

Paired *t*-tests showed that valence ratings did not significantly differ between sad (4.29 ± 1.46) and happy music (5.21 ± 0.88), *P* = 0.013 (Bonferroni-adjusted alpha level of .012), suggesting that sad music also was associated to some extent with pleasurable emotional experiences. Similarly, arousal ratings did not significantly differ between sad (3.21 ± 1.18) and happy music (3.75 ± 0.9), *P* = 0.04 (Bonferroni-adjusted alpha level of 0.012), in accordance with the use of music stimuli controlled for tempo characteristics. Furthermore, sadness ratings were significantly higher during sad (4.54 ± 0.83) compared with happy music (1.5 ± 0.83), *t*(23) = 10.90, *P* < 0.001. Inversely, happiness ratings were significantly higher during happy (5.42 ± 0.72) compared with sad music (2.71 ± 1.27), *t*(23) = 8.74, *P* < 0.001.

### fMRI results

**Fig. 1.**
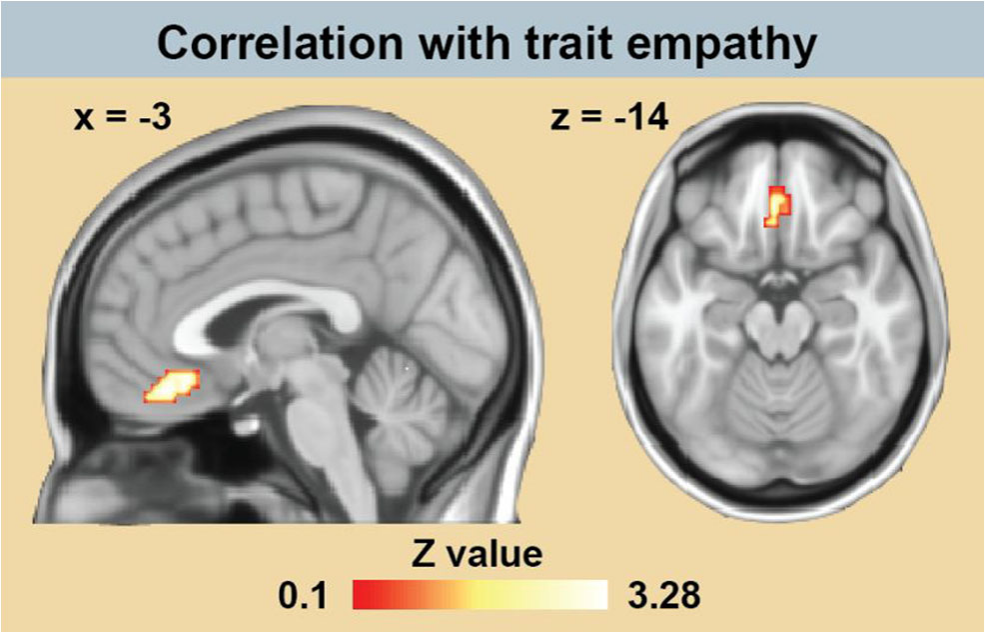
Results of the correlation analysis between eigenvector centrality maps and empathy scores. Positive correlations (shown in red-yellow colors) were found in a cluster located in the ventromedial prefrontal cortex, including inferiorly part of the orbitofrontal cortex. Results were controlled for age and gender, and corrected for multiple comparisons (*P* < 0.05). Coordinates refer to MNI space.

**Fig. 2.**
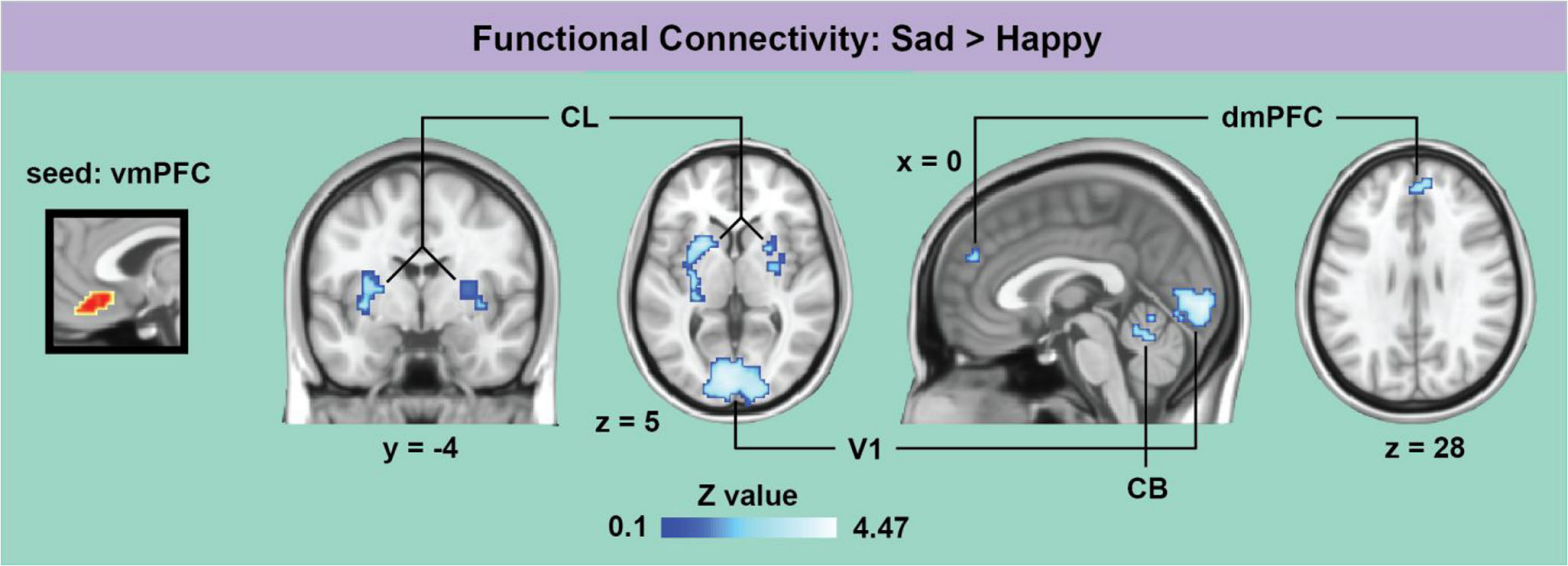
Results of the comparison of functional connectivity maps between the sad and happy condition (*sad > happy*). The cluster located in the ventromedial prefrontal cortex (vmPFC) extending to the medial orbitofrontal cortex showed stronger functional connectivity with the dorsomedial prefrontal cortex (dmPFC), primary visual cortex (V_1_), bilateral claustrum (CL)/putamen, and cerebellum (CB). Results were corrected for multiple comparisons (*P* < 0.05). Coordinates refer to MNI space.

Significant positive correlations between EC and the total empathy scores were observed in a cluster of voxels located in the vmPFC, including (but not restricted to) the mOFC (Fig. 1; Table 1), suggesting that the vmPFC is more crucial to emotional processes in people with high empathy scores. This EC cluster exhibited significantly stronger functional connectivity during sad than during happy music with the dmPFC, primary visual cortex (V_1_), bilateral claustrum (CL), putamen (PT), and cerebellum (CB) (Fig. 2; Table 1). The V_1_ exhibited the strongest connectivity and was by far the largest target region identified (Table 1). Conversely, no region was found to show significantly stronger functional connectivity with the vmPFC/mOFC during happy compared with sad music.

**Table 1.** Results of empathy correlation and functional connectivity analyses for the contrast *sad > happy*, corrected for multiple comparisons (*P* < 0.05)

Anatomical location	MNI coordinates	Cluster size (mm^3^)	*z*-value: max (mean)
Empathy correlation			
vmPFC/mOFC	-3 36 -14	1,053	3.28 (2.75)
FC (seed region: vmPFC/mOFC)			
dmPFC	9 48 37	972	3.09 (2.78)
L. CL/PT	-24 12 10	5,238	3.77 (2.93)
R. CL/PT	27 -6 10	1,404	3.35 (2.79)
CB (lobule V, 70%)	-3 -60 -5	1,026	3.46 (2.84)
Calcarine sulcus (V_1_, 90%)	-6 -90 1	15,255	4.47 (3.11)

Outermost right column shows the maximal *z*-value within a cluster (with the mean *z*-value of all voxels within a cluster in parentheses). Percentage in parentheses indicates the anatomical probability according to the SPM Anatomy Toolbox (Eickhoff et al., 2005). CB = cerebellum; CL = claustrum; dmPFC = dorsomedial prefrontal cortex; mOFC = medial orbitofrontal cortex; PT = putamen; V_1_ = primary visual cortex; vmPFC = ventromedial prefrontal cortex.

## Discussion

This study explored how dispositional empathy modulates neural responses to sad (compared with happy) music. Previous behavioral investigations highlighted that the experience underlying listening to sad music is highly modulated by trait empathy, which leads to variability in emotional valence. Using ECM in combination with FC, we demonstrate that individual differences in trait empathy are associated with higher centrality within a distributed network of brain areas encompassing vmPFC/mOFC, dmPFC, V_1_, CL/PT, and CB, which are engaged while listening to sad (vs. happy) music. The vmPFC/mOFC acts as a “computational hub” and the remaining brain areas as functionally connected nodes.

In accordance with our hypothesis, the ECM results revealed that empathy scores correlated with centrality values in the mOFC (part of a larger cluster covering the vmPFC). The mOFC has been recently indicated as core hub of the compassion network (Singer & Klimecki, 2014). Specifically, activations of the mOFC have been reported in meditation-naïve participants who, after following a short-term compassion training, were exposed to short film excerpts depicting human suffering (Klimecki et al., 2013). Furthermore, patients with damage in the vmPFC exhibit impaired empathy, poor decision making, and a deterioration of “moral character,” because they are unable to generate the feelings that guide adaptive decision making in healthy individuals (Anderson et al., 1999; Bechara et al., 1996; Shamay-Tsoory, 2007). Similarly, activations of the vmPFC have been previously related to affective empathy (Hynes et al., 2006; Mobbs et al., 2009; Saxe, 2006; Völlm et al., 2006), in particular to empathy for positive emotions (Morelli et al., 2014). In light of these previous findings, the observed data suggest that listeners with a predisposition to empathize took a compassionate, rather than distressed, stance toward sad music. This is in line with (*i*) the observed high centrality in two bilateral clusters encompassing the PT (Table 1), another core region of the compassion network (Klimecki et al., 2012), and (*ii*) the fact that we did not observe activity in regions typically involved in empathy for pain (Jackson et al., 2005; Lamm et al., 2011; Singer et al., 2004). Notably, our finding ties in well with the music-and-emotion literature revealing that enjoyment of sad music is positively correlated with trait empathy (Garrido & Schubert, 2011; Kawakami & Katahira, 2015; Taruffi & Koelsch, 2014; Vuoskoski, et al., 2012). It is important to mention that, in this study, the observed centrality of the vmPFC/mOFC was not a result of familiarity effects with sad music (as in Wallmark et al., 2018). Sad and happy stimuli were in fact controlled for familiarity and participants were equally unfamiliar to both emotion conditions (see *Methods*).

We obtained also evidence for the involvement of the dmPFC, which exhibited functional connectivity with the vmPFC/mOFC during sad compared with happy music. The dmPFC plays a pivotal role in mentalizing (e.g., Amodio & Frith, 2006), and specifically in the attribution of enduring traits and qualities about others (Van Overwalle, 2009). Interestingly, activity in the dmPFC predicts altruistic behavior (Moll et al., 2006; Waytz et al., 2012), consistent with the view that prosocial tendencies rely on the capacity to understand the minds of others. In line with our result, a previous music study found that trait empathy correlates with activity in the mPFC during listening to familiar versus unfamiliar music (Wallmark et al., 2018). Therefore, the observed functional connection between the vmPFC/mOFC and dmPFC may suggest that empathic participants engaged with mentalizing-related computations, such as, e.g., fantasizing about other people or fictional characters to undergo imagined events. Levinson (1997) has previously argued that experiencing music as a narrative is one compelling way through which listeners empathize with the music (another common music-empathy mechanism is *emotional contagion*, where the listener internally mirrors the perceived emotional expression of music by means of physiological feedback of muscular and autonomic activity; Juslin & Västfjäll, 2008; Lundqvist et al., 2009). This mechanism of cognitive empathy in music is similar to what readers do with a novel's fictional character (Tamir et al., 2015), but with the difference that listeners can imagine their own narrative unfolding on the basis of the musical events. Notably, simulation of other people’s minds during reading is crucially supported by the default mode network and in particular by the dmPFC, which responds preferentially to passage with social and abstract content (Tamir et al., 2015).

The observed functional connection between vmPFC and V_1_ during sad compared with happy music is in line with our hypothesis, suggesting an association between visual mental imagery processes and listening to sad music, although the causal direction of this relationship requires further investigation. Neuroimaging literature has provided clear evidence of a large overlap between visual perception and visual imagery. Specifically, the early visual cortex (V_1_) supports the construction of visual mental images (Kosslyn & Thompson, 2003). This parallel between cognitive resources involved in imagery and perception also largely applies to the other sensory modalities (Kosslyn et al., 2001). Music very often stimulates internal images in the listener (Küssner & Eerola, 2019), consisting of pictorial representations (natural landscape, colors), embodied image-schemata (picturing a melodic movement as an ascending or descending image), or complex visual narratives (similar to a movie) (Taruffi & Küssner, 2019). Sad music has been found to trigger enhanced mind-wandering in the form of visual mental imagery with emotion- and nature-related content (Taruffi, Pehrs, et al., 2017). Moreover, previous fMRI studies of music and emotion reported the engagement of the primary and secondary visual cortices during music listening (Koelsch & Skouras, 2014; Trost et al., 2012; Wallmark et al., 2018). In these experiments—as in the current one—participants underwent scanning with their eyes closed. Therefore, the present data support our hypothesis that individuals who are prone to empathize exhibit enhanced activity of V_1_ and that this pattern is more pronounced during listening to sad compared with happy instrumental film music. Importantly, of the observed functionally connected structures, V_1_ exhibited the highest centrality values and was by far the largest region, suggesting that visual mental imagery might be a central mechanism underlying empathic individuals’ responses to sad music. Furthermore, these findings lead to the intriguing hypothesis to be tested by future research that experiencing vivid visual mental imagery may facilitate empathic participants to transpose themselves into the feelings and thoughts of their imagined characters or events during the music.

Of particular interest is the functional connection between the vmPFC and bilateral CL during sad compared with happy music. The CL—whose function has remained rather obscure to date—is a thin, irregular sheet of gray matter that lies below the general region of the insular cortex and above the PT (Crick & Koch, 2005). The CL has extensive reciprocal connections to almost all cortical areas and also to a number of subcortical areas, including lateral amygdala, caudate, PT, and globus pallidus (Fernandez-Miranda et al., 2008; LeVay & Sherk, 1981; Park et al., 2012). Due to these widespread connections, Crick and Koch (2005) proposed that the CL synchronizes and binds separate multisensory information, including perceptual, cognitive, motor and emotional content, to form a unitary, single object, thus serving as a consciousness center for the brain. This proposal is consistent with a number of neuroimaging studies showing the involvement of the CL in tasks in which integration of multimodal information is required (Banati et al., 2000; Baugh et al., 2011). Changes in claustral activity in neuroimaging studies of music and emotion are rather uncommon; however, this may be due to the small size of the CL and its proximity to the insula (i.e., instead more commonly engaged during music listening; see, for example, Caria et al., 2011), which make it challenging to discriminate between claustral and insular activity. The functional connectivity between vmPFC and CL observed in the present study is consistent with anatomical bidirectional projections from the CL to the PFC (Park et al., 2012; Tanné-Gariépy et al., 2002) and suggests a role of the CL in the integration of the different affective, perceptual, and cognitive processes underlying listening to sad music. Regarding the functional connection between vmPFC and CB, changes in cerebellar activity have been previously linked to dispositional empathy (Jackson et al., 2005; Moriguchi et al., 2007; Singer et al., 2004). In particular in the study by Singer et al. (2004), individuals scoring higher on empathy (as measured by the empathic concern subscale of the IRI and the Balanced Emotional Empathy Scale from Mehrabian & Epstein, 1972) showed higher pain-related activity in ACC, left AI, and also lateral right cerebellum. Another possible interpretation could be related to rhythmic entrainment. The cerebellum is involved in the neural tracking of rhythm (e.g., Nozaradan et al., 2017), and trait empathy is positively associated with (sensorimotor) rhythmic entrainment abilities (e.g., Bamford & Davidson, 2019).

In the broad context, our findings extend the current understanding of empathic behaviors to the musical, and in general aesthetic, domain. In line with the previous account of compassion as a social emotion characterized by a concern for another person’s suffering, which is accompanied by positive feelings and a motivation to help (Singer & Klimecki, 2014), our study shows that trait empathy is correlated with centrality values within brain regions (mOFC and PT) that are crucially involved in the generation of compassionate feelings also in response to music—an abstract stimulus that does not contain any explicit reference to a human agent. In addition, in our study empathic participants exhibited enhanced centrality values in brain regions that typically underlie social cognition (dmFPC) and mental imagery (V_1_). Overall, these centrality patterns were specific for sad music, suggesting that this type of music may constitute an effective social signal that triggers specific empathy-related processes in individuals who are already prone to empathy. Although we did not find any significant result for the contrast happy versus sad, our findings do not exclude the possibility that the observed patterns of centrality during sad music also may be at play during happy music. People may find it easier to engage with happy rather than sad music, given that the latter represents a more complex emotion with positive and negative nuances, which could be more readily available to empathic individuals who are prone to transfer themselves into others’ emotions and perspectives; such difference may consequently lead to a range of more sophisticated or enhanced neural responses to sad music in high-empathy participants (e.g., integrating complex affective and cognitive processes), as suggested by the present study.

Furthermore, our findings underscore the importance of considering individual differences when investigating the neural mechanisms underlying musical experiences. This is particularly relevant for the case of sad music, where individual characteristics play a key role. A piece of sad music can be associated with feelings of sorrow or be experienced as pleasurable by someone else. Therefore, future neuroimaging research should foster experimental paradigms that take into account the variance brought about by individual differences in emotional responses to music. Although previous accounts of music-empathy focused predominantly on emotional contagion (e.g., Davies, 2011; Juslin & Västfjäll, 2008; Lundqvist et al., 2009), our study points to more sophisticated forms of empathy, involving compassion, mentalizing, and fantasy processes. Clearly, this could be due to the particular music genre of the stimuli employed in this study—film soundtrack—and a question arises about the extent to which the current results can be generalized to other music genres. Film music is in fact particularly effective in facilitating listeners to conjure up visual images and to evoke intense emotions as well as vivid daydreams. Therefore, this issue should be addressed by future research employing stimuli from other music genres.

In future studies, it would be important to show how in empathic individuals the observed neural activity pattern maps onto behavioral responses to sad music. Our data along with evidence collected by previous studies (Eerola et al., 2016; Garrido & Schubert, 2011; Kawakami & Katahira, 2015; Taruffi & Koelsch, 2014; Vuoskoski & Eerola, 2012; Vuoskoski et al., 2012) suggest that empathic individuals experience more complex emotional responses toward sad music, including both positive and negative facets. However, this needs to be validated by using a more thorough behavioral assessment in the scanner. Nevertheless, our study still provides an intriguing step forward in the scholarship concerning empathy and sad music by showing that a unique network of brain regions related to individual differences in trait empathy is significantly more active for sad music than for happy music. Finally, although we estimated our target sample on previous research that successfully identified neural correlates of emotional personality using music (Koelsch et al., 2013), we underline here the necessity for future research to increase sample size. Because our sample is relatively small in the context of individual differences research, it could be possible that more potential results have been missed.

## Conclusions

This study identified a distributed brain network associated with individual differences in trait empathy and spontaneously recruited during listening to sad music. This “music-empathy” network comprises brain regions subserving coding of compassion, mentalizing, and visual mental imagery. In addition, a novel site of activation was found in the CL, showing its involvement in a music listening task. Our study contributes to the increasingly sophisticated understanding of the empathic brain, mapping neural dynamics to specific individual characteristics. In this sense, our results are promising because they suggest that the variation in brain-network connectivity provides a valid marker of empathic abilities. Moreover, the fact that music acts as a social stimulus—by triggering empathic individuals to undergo enhanced emotional and cognitive experiences—opens novel possibilities, involving the use of music tasks, for social neurosciences and, in general, speaks about the importance of empathy in aesthetic contexts (an issue that has been discussed by philosophers but mostly overlooked by neuroscientists).
